# Effects of Different Ion Irradiation on the Contact Resistance of Pd/Graphene Contacts

**DOI:** 10.3390/ma12233928

**Published:** 2019-11-27

**Authors:** Kashif Shahzad, Kunpeng Jia, Chao Zhao, Dahai Wang, Muhammad Usman, Jun Luo

**Affiliations:** 1 Key Laboratory of Microelectronic Devices & Integrated Technology, Institute of Microelectronics, Chinese Academy of Sciences, Chaoyang District, Beijing 100029, China; kashif@ime.ac.cn (K.S.); jiakunpeng@ime.ac.cn (K.J.); wangdahai@ime.ac.cn (D.W.); 2 School of Microelectronics, University of Chinese Academy of Sciences (UCAS), Beijing 100031, China; 3 National Center for Physics, Quaid-i-Azam University, 46000 Islamabad, Pakistan

**Keywords:** contact resistance (R_c_), graphene, ion-irradiation, transmission line method (TLM)

## Abstract

The effect of ion-induced defects on graphene was studied to investigate the contact resistance of 40 nm palladium (Pd) contacting on graphene. The defect development was considered and analyzed by irradiating boron (B), carbon (C), nitrogen (N_2_), and argon (Ar) ions on as-transferred graphene before metallization. The bombardment energy was set at 1.5 keV and ion dose at 1 × 10^14^ ions/cm^2^. The defect yields under different ion irradiation conditions were examined by Raman spectroscopy. Although, dissolution process occurs spontaneously upon metal deposition, chemical reaction between metal and graphene is more pronounced at higher temperatures. The rapid thermal annealing (RTA) treatment was performed to improve the Pd/graphene contact after annealing at 450 °C, 500 °C, 550 °C, and 600 °C. The lowest contact resistance of 95.2 Ω-µm was achieved at 550 °C RTA with Ar ion irradiation. We have proved that ion irradiation significantly enhance the Pd/graphene contact instead of pd/pristine graphene contact. Therefore, in view of the contention of results ion induced defects before metallization plus the RTA served an excellent purpose to reduce the contact resistance.

## 1. Introduction

Graphene has been studied lately during the course of establishing high-speed radiofrequency devices, interconnects [1], photonics [2,3], and flexible electronics [4]. By now the main hindrance is the reported contact resistance for graphene compared to silicon devices. In order to reduce the contact resistance, different methods have been used, ranging from the cleaning the graphene surface to the use of different metals and annealing at different temperatures. Therefore, in order to improve the carrier transmittance and reduce contact resistivity, it is necessary to study the monoatomic graphene layer, which is completely different from typical silicon junctions. 

The contact resistance (R_c_) values are currently determined by the chemical bonds, interface engineering, and electronic structure of graphene. The challenge which poses a barrier towards integration and reduction of graphene electronic devices is the reproducible formation of low contact resistance. However, although contact resistance is an important obstacle for further improvement, which is still not well understood. Conventional deposition of metal electrodes on top of the graphene surface often resulted in a large contact resistance [5,6,7]. In order to modify the Fermi level difference between metal and graphene to improve the metal/graphene (M/G) interface, distinct metals with different work functions were initially attempted [8]. Several articles report the increase in the density of states (DOS) in graphene and decrease in contact resistance for surface treatments or innovative device architecture [9,10]. 

To reduce the contact resistance scientists have adopted different techniques to reduce the contact resistance by controlling the M/G interface fabrication and improving its structures. These techniques are believed to be the ideal solution to address the problem of contact resistance by fabricating clean M/G interface. For this purpose, the fabrication process was modified by introducing the aluminum (Al) cap layer between graphene and photoresist to hold back the resist residues to spread over the surface [11]. The interaction of residual polymers during surface transfer makes the Fermi level shift, which leads to the decrease of carrier mobility and the contamination of the M/G interface, resulting in an increase of R_c_. [12,13,14,15,16]. During the lithography process before metal deposition, ozone/ultraviolet treatment is used for cleaning to improve M/G contact prior to metallization [17,18]. The basic principle of these methods is to minimize the organic residues in the metal contact zone of graphene devices by photolithography.

The large contact resistance of graphene is due to the lack of surface bonding sites, resulting in the lack of chemical bonds and strong orbital hybridization [19,20,21,22]. Therefore, the modification of the contact region has been realized by different research groups. The substantial decrease in contact resistance is observed by parallel cutting of graphene [6,20]. In order to obtain low contact resistance, the formation of edge contact geometry was studied, and the hole diameters between 50 and 1000 nm were patterned [23]. Oxygen plasma treatment duration has been increased up to 65 sec to replace the C-C bond with the C-O bond, the edge contacts with oxygen termination enhance the M/G interface [24,25]. Electron beam lithography is also used to achieve a lower R_c_ value by producing an array of antidots in the patterned region of graphene [26]. Edge contacts are considered as the novel method to realize graphene metal contact [5,6,24,27,28]. Although the important advantages of realizing the end contact structure of graphene have been predicted theoretically, it is an arduous task from the experimental point of view. Furthermore, dissolution of carbon requires initiation sites like defects or dangling bonds in graphene. The application of different patterns or edge contact through different development techniques is too complicated to achieve. Based on this knowledge, a method with a reduced number of fabrication steps and minimum involvement of photoresist developments is imperative. 

In this paper, the formation of end contact by ion irradiation is proposed. A good strategy for forming end contact in planar device structures is to increase the exposure of graphene defects in source and drain areas before metallization. In this work an elegant approach featuring the concept of end contact is developed to improve metal graphene interface. The density of defects is controlled by the ion dose. In order to understand the formation of defects and their effect on contact resistance, it is necessary to understand the defect yield at different energies and doses. The functionalization of the surface of graphene is done at pre-selected areas of graphene to increase the chemical reactivity of the metal contact deposited on graphene and forms end contacts. Another important feature of this technique is to improve the conductivity across the metal graphene interface by enhancing the adhesion of metal and graphene through defects. However, the density and size of defects may vary with irradiation of different ion species. Moreover, the main point of this research work is to improve the metal/graphene interface. This consequently enables us to achieve low contact resistance of palladium (Pd) metal contact. The effect of irradiation of different ion B, C, N_2_ and Ar on the contact resistance of graphene with Pd as the contact metal at 1.5 keV energy has been studied in this paper. In this study, Pd is chosen as the electrode material, because Pd is a chemosorption metal. It is theoretically predicted that it will react strongly with graphene through orbital hybridization and always provide low resistance contact for graphene. Rapid thermal annealing was carried out at 450 °C, 500 °C, 550 °C, and 600 °C. In addition, R_c_ values were compared before and after rapid thermal annealing (RTA).

## 2. Materials and Methods 

**Figure 1:**
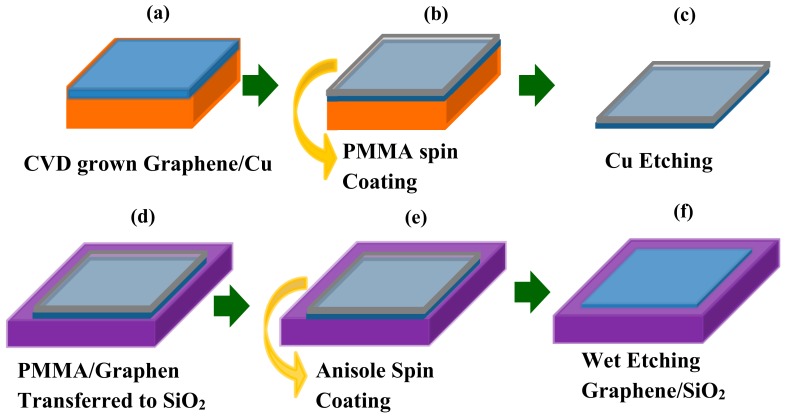
Schematics of the transfer process; (**a**) chemical vapor deposition (CVD) grown graphene on Cu, (**b**) a film of PMMA is spin coated as protective layer, (**c**) then the Cu foil is etched, (**d**) PMMA/graphene stack transferred to SiO_2_ substrate, (**e**) anisole spin coating, and (**f**) the polymer is dissolved leaving graphene surface free.

**Figure 2:**
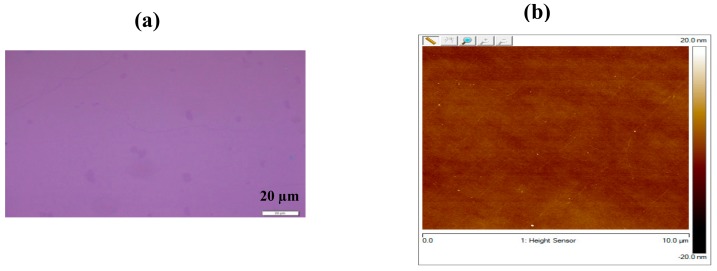
(**a**) optical image and (**b**) atomic force microscopy (AFM) image of as transferred graphene on to the surface of SiO_2_.

To describe the contact treatment process, chemical vapor deposition (CVD) grown graphene was first transferred to 300 nm highly p-doped SiO_2_ substrate. The graphene samples used in this work were grown on 25 µm thick Cu foil by CVD. Polymethyl-methacrylate (PMMA) (950 K, 4.5% in anisole) was spin-coated at 500 rpm for 5 s and 3000 rpm for 50 s, on the CVD grown graphene as a shield to protect from damage during the transfer process. The samples left in the clean atmosphere for 15 min to solidify the PMMA film. The PMMA/graphene/Cu samples were then placed in cleansing solution (HCl:H_2_O_2_:H_2_O = 1:1:1) for 50 s to remove the dust and the residues attached on back side of Cu during the spin coating. The accessible graphene-copper face was then etched by immersing in a marble solution HCl (50 ml): H_2_O (50 ml): CuSO_4_.5H_2_O (10 g) for 1.5 h, leaving behind a pliant membrane of PMMA/graphene suspended in the solution. Cu etching residues are cleaned by rinsing in Deionized (DI) water five times. The floating flexible and fragile membrane was transferred on the SiO_2_ substrate (300 nm SiO_2_ grown on Si substrate) with care and precision. The PMMA layer is then removed by rinsing in acetone 3 times for 5 min each and then in ethyl alcohol three times for 5 min each. Then finally the samples are rinsed in DI water five times. The surface morphology of transferred graphene was investigated by optical spectroscopy, atomic force microscopy (AFM, IMECAS, Beijing, China), and Raman spectroscopy. Figure 1 shows the schematic diagram of a CVD grown graphene transfer process. The process was carried out in the cleanroom. Figure 2 shows the optical and AFM image of as transferred graphene. The successful transfer of graphene was confirmed by Raman spectroscopy (Institute of Physics CAS, Beijing, China). The AFM and optical spectroscopy were performed to ensure the clean transfer of graphene onto the substrate. The AFM is used to characterize the surface roughness and the maximum height (R_max_) of the large residue particles on the surface of as-transferred graphene. R_max_ is the maximum height of the polymer residue particles generated during transfer. These two parameters are important for the metal/graphene interface contact resistance. The surface roughness and R_max_ measured in our case are 1.3 nm and 33 nm, respectively. Moreover, the measured values confirm the clean transfer of graphene film. Transfer length method (TLM) test structures were fabricated to determine contact resistance between graphene and metal. To further prevent the graphene surface from photoresist residues a sacrificial layer of Al_2_O_3_ was laid down on to the graphene as a hard mask using atomic layer deposition.

**Figure 3:**
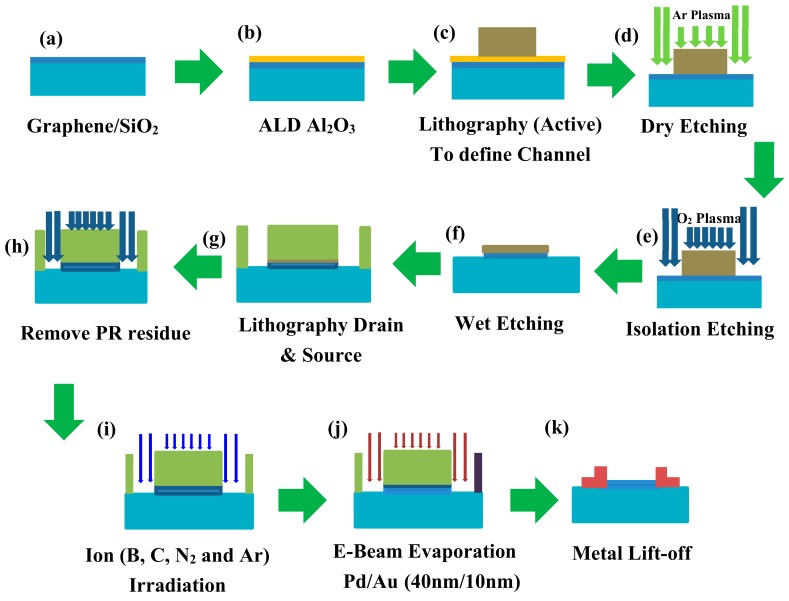
Schematics of fabrication process flow of transfer length method for contact resistance measurement. (**a**) CVD grown graphene transferred onto SiO_2_, (**b**) the deposition of Al_2_O_3_ isolation layer by atomic layer deposition (ALD), (**c**) definition of graphene channel by first lithography, (**d**) Ar plasma etching for Photo-resist (PR) residues removal, (**e**) graphene etching by O_2_ plasma, (**f**) wet etching of photoresist, (**g**) contact window opening by second lithography, (**h**) PR removal by Ar plasma, (**i**) ion (B, C, N_2_, and Ar) irradiation on graphene at contact window, (**j**) metal deposition by e-beam evaporation, and (**k**) lift-off process of unwanted metals and photoresist.

Figure 3 shows the schematics of the detailed fabrication of the TLM test structures. The positive photoresist was used to protect the graphene channel, while argon plasma is used to remove the unwanted Al_2_O_3_ and graphene. Contact windows for metal deposition were defined by another lithographic process using negative photoresist. The protective layer of Al_2_O_3_ was removed across the contact window by wet etching using dilute solution of H_3_PO_4_ at 40 °C for 12 min. The samples have been further divided and classified into four different categories. Each sample was irradiated with different ion species of B, C, N_2,_ and Ar at energy of 1.5 keV and a dose of 1 × 10^14^ ions/cm^2^ on the specified pre-selected contact area of graphene. The energy and ion dose of each group remained unchanged. Furthermore, ion irradiation was only done on the graphene surface where contact metal is to be deposited. The channel lengths remain unaffected by ion irradiation. The contacted metal Pd was deposited on to the samples by e-beam evaporation. The metal lift-off process was carried out by immersing the samples in acetone three times and then in alcohol twice, for 5 min each. To investigate the metal graphene contact improvement after annealing, RTA is performed at different temperatures i.e., 450 °C, 500 °C, 550 °C, and 600 °C. The RTA was done in atmosphere of N_2_ for 1 min. The contact resistance measurements were performed before and after RTA by using Keithley 4200 semiconductor parameter analyzer (IMECAS, Beijing, China) at room temperature and pressure conditions. 

## 3. Results

**Figure 4:**
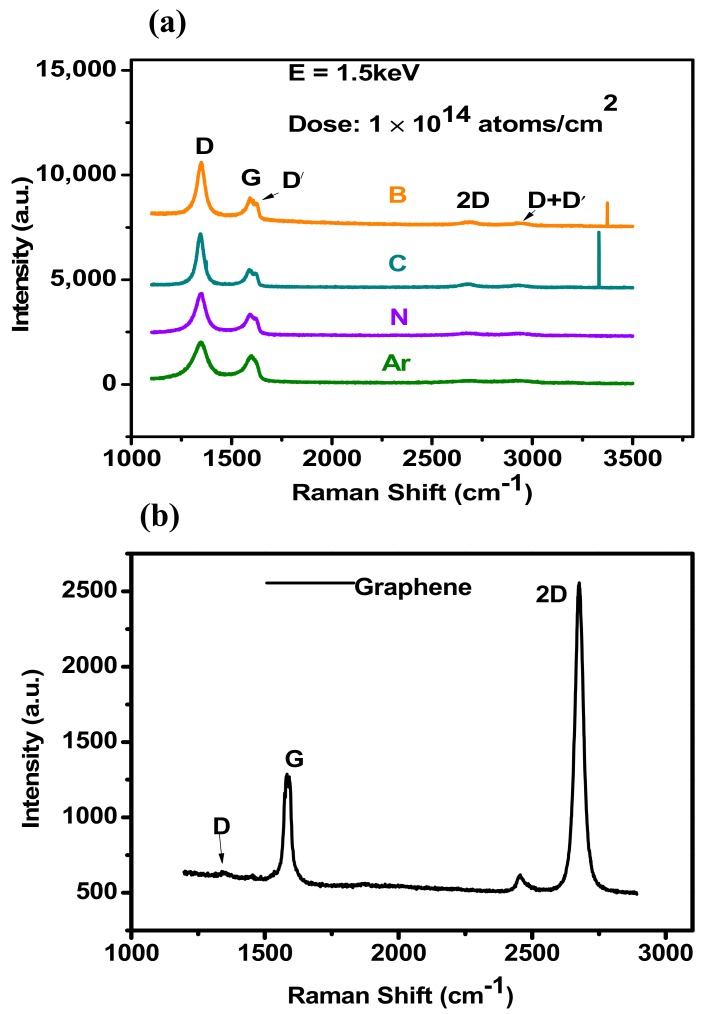
(**a**) Raman spectra of as-transferred graphene on SiO_2_ substrate, obtained by irradiation of B, C, N_2_, and Ar ions for energy of 1.5 keV at the dose of 1 × 10^14^ ions/cm^2^. (**b**) Raman spectra of CVD grown graphene transferred to SiO_2_.

Figure 4 shows Raman spectra of as-transferred graphene on to the SiO_2_ substrate, the two intense features, one-phonon mode G line originating from the center of Brillion zone centered at about 1560 cm^−1^ and a two-phonon mode called 2D line originating from the K point of Brillouin zone at 2700 cm^−1^, with the I_2D_/I_G_ ratio > 2, prior to irradiation [29]. The spectrum adduced from the 532 nm excitation laser at room temperature and pressure conditions. With the absence of D peak in the spectrum, the intensity ratio and sharpness of 2D and G peaks describe the clean transfer of monolayer graphene sheet to the substrate and absence of induced observable defects. Therefore, the negligible D-band signal confirms transfer process was carried out successfully with a smaller number of defects. In short, this does attest the fact that the defect evolution started after ion irradiation on to the surface of transferred graphene. 

**Figure 5:**
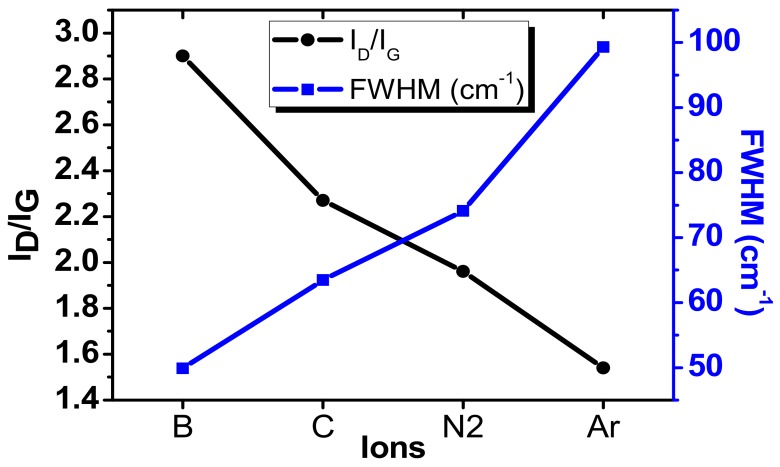
Ratio of D to G peak I_D_/I_G_ and full width at half maxima (FWHM) of D-peak of ion irradiated graphene for ions B, C, N_2_, and Ar.

Figure 4 shows the Raman spectrum of the samples irradiated with different ion species i.e., B, C, N_2_, and Ar, at an ion dose of 1 × 10^14^ ions/cm. The energy of the ion species was set at 1.5 keV. As can be seen, the significant change in the Raman spectrum of irradiated samples was observed. The laser excitation activated two extra lines called D and D′ in the samples irradiated with B, C, N_2_, and Ar ions. Irrespective of defect-free Raman spectra of pristine graphene defined by G and 2D bands, the zone boundary phonons give rise to the strong signal peak at ~1350 cm^−1^ known as D peak. The single and sharp D-peak appears in the case of B and C ion irradiated samples. This implies that the samples subjected to ion irradiation showed disordered surface with induced defects. Therefore, the ion irradiation contributed to the disordered Raman induced signature. Furthermore, broken C-C bond also gives rise to D-band signal. The full width at half maxima (FWHM) of D peak is lowest in case of B, while the maximum value achieved in case of the samples irradiated with Ar, the values are shown in Figure 5. 

In addition, the emergence of D′ peak occurs as a shoulder to G peak in case of N_2_ and Ar ion irradiation. However, the D′ peak did not appear in case of B and C ion Irradiation. The 2D band signal gradually decreased, mainly due to the lattice vibration mode suppression corresponding to the 2D peak caused by defects for all the samples after irradiation exposure. The D/G ratio after exposure increases, thus structurally defected area increases. These changes in Raman spectra indicate the progressive disorder of graphene structure with defect density. However, this evolution is attributed to the irradiation of different ions at 1.5 keV energy. The D peak appeared in the spectrum after exposure to irradiation showed different intensities.

Figure 5 shows the summary of experimentally determined D/G values for the ions B, C, N_2_, and Ar irradiated on to the graphene samples. To compare the number of defects for different ion species the Raman data is normalized with the intensity of G peak I (G). As the energy and dose remain persistent for different ion species used here, the intensity of the D peak is different for different ions. The intensity and FWHM of D and G peaks defy the amorphous nature of graphene. However, the vacancy type defects are dominating for each ion irradiated graphene sheet. The maximum I_D_/I_G_ is obtained in the case of B ions, while the lowest value is claimed in case of Ar. The D′ and G peak started to merge in the case of N_2_ and Ar. However, the intensity starts to decrease and FWHM starts to increase. Thus, it is difficult to determine the separate contribution of individual D′ and G peaks. Therefore, as the ion size increases the size of defect increases and inter distance between defects also decreases. The main reason for this is the energy transfer mechanism of incident ions, where the electronic and nuclear stopping is proportional to the mass of ion. Therefore, the defect yield depends on the ion dose and ion species. Moreover, the defect formation at prescribed energy is due to nuclear collisions, the electronic stopping contribution is ignored at 1.5 keV. By measuring Raman signals at 1.5 keV with different ion species, we believe that the defect generation rate is not only related to ion dose, but also to the ion species and irradiation energy. In general, the total defect production is proportional to the ion dose and energy. 

**Figure 6:**
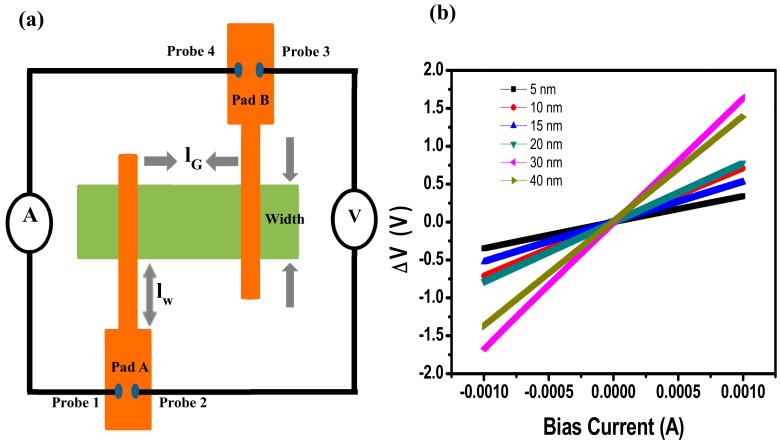
(**a**) two probe measurement configuration and (**b**) measured I–V characteristics of M/G contact for different l_G_ in transfer length method (TLM) structure.

Figure 6a shows the two-probe contact resistance measurement arrangement employed here. The biased current is supplied across probe one and probe four, while the corresponding change is potential is measured across probe two and probe three. Figure 6b shows the I–V characteristic curves of typical graphene-metal contact at different lengths of graphene in TLM structure, which confirms the establishment of good ohmic contact after RTA. The total resistance (R_T_) measured in this process by four probe method is elaborated by the following equation.

$$R_{T}=R_{s}\left(l_{g}\right)+2R_{GM}+R_{w}\left(l_{w}\right)+2R_{pp}$$

where *R_T_* is the total resistance, which depends on the *R_S_* sheet resistance of graphene, metal wire resistances *R_w_* and graphene metal contact resistance *R_GM_*, and probe pad configuration resistance *R_pp_*. The use of four probes provides the advantage in which probe pad resistance can be ignored. Metal wires deposited in our case are thin and long enough to ignore their contribution. Therefore, their contribution to the total resistance is subtracted to get a linear relationship.

**Figure 7:**
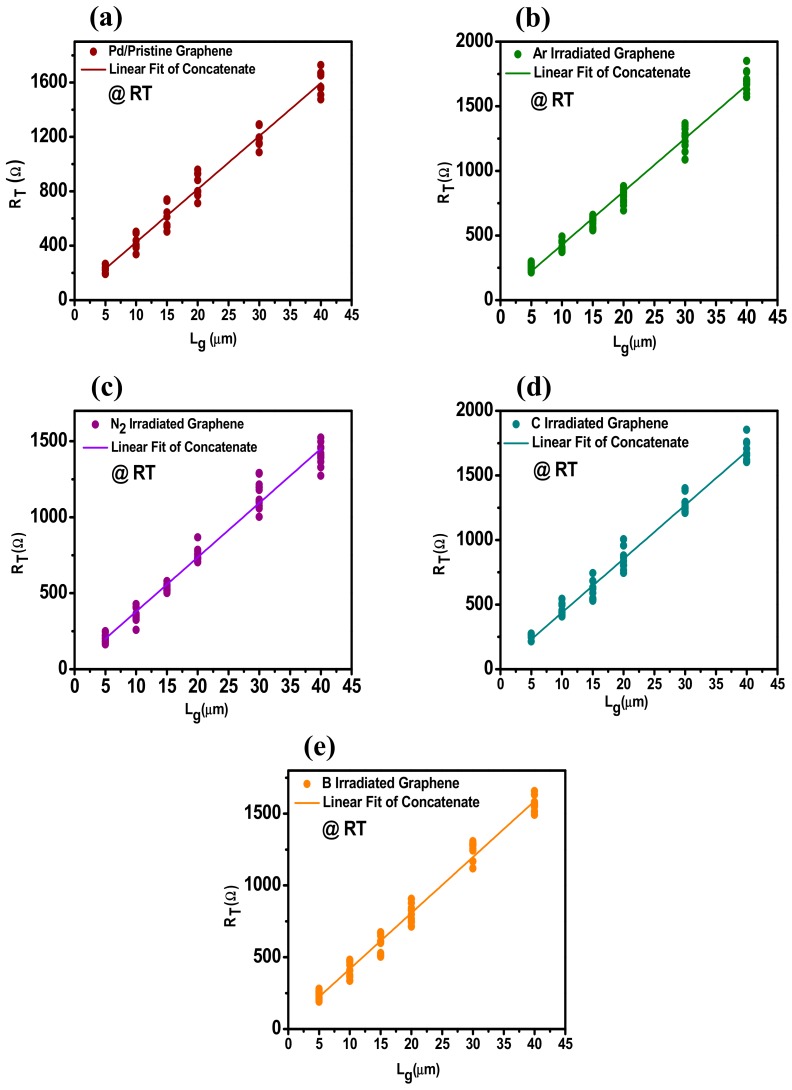
Linear plots of total resistance R_T_ versus length of graphene l_G_ for contacts of B, C, N_2_, and Ar irradiated induced defects on graphene, with Pd/Au (40 nm/10 nm) contacts at room temperature (RT), 10 individual measurements were used at each channel length (l_G_) and the linear fitting was performed using a concatenate fit method; (**a**) linear fitting of Pd/Au (40 nm/10 nm) on pristine graphene at RT, (**b**) linear fitting of Pd/Au (40 nm/10 nm) on Ar irradiated graphene at RT, (**c**) linear fitting of Pd/Au (40 nm/10 nm) on N_2_ irradiated graphene at RT, (**d**) linear fitting of Pd/Au (40 nm/10 nm) on C irradiated graphene at RT, and (**e**) linear fitting of Pd/Au (40 nm/10 nm) on B irradiated graphene at RT.

**Figure 8:**
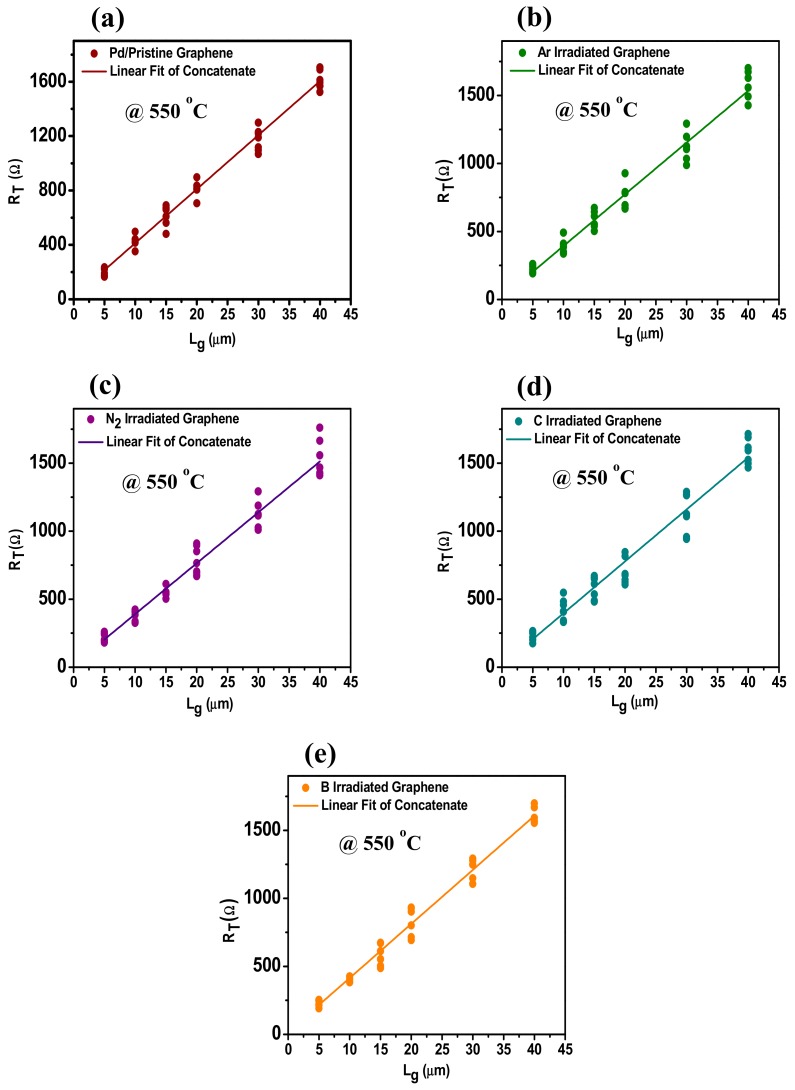
Linear plots of total resistance R_T_ versus length of graphene l_G_ for contacts of B, C, N_2_, and Ar irradiated induced defects on graphene, with Pd/Au (40nm/ 10nm) contacts at 550 °C rapid thermal annealing (RTA), 10 individual data points were used at each l_G_ and the linear fitting was performed using a concatenate fit method; (**a**) linear fitting of Pd/Au (40 nm/10 nm) on pristine graphene at 550 °C RTA, (**b**) linear fitting of Pd/Au (40 nm/10 nm) on Ar irradiated graphene at 550 °C RTA, (**c**) linear fitting of Pd/Au (40 nm/10 nm) on N_2_ irradiated graphene at 550 °C RTA, (**d**) linear fitting of Pd/Au (40 nm/10 nm) on C irradiated graphene at 550 °C RTA, (**e**) linear fitting of Pd/Au (40 nm/10 nm) on B graphene at 550 °C RTA.

For the contact between Pd and graphene, the linear plots of R_T_ versus channel length (l_G_) of samples irradiated by different ions are shown in Figure 7 at room temperature. While the Figure 8 shows the R_T_ versus l_G_ of the samples treated at 550 °C. The TLM configuration was employed here to measure the contact resistance. The contact resistance of ion-irradiated graphene Pd contact before and after RTA has been measured by four-probe measurement structure, at temperatures of 450 °C, 500 °C, 550 °C, and 600 °C. The same measurements were performed after each RTA step. Figure 7 and Figure 8 show the linear relationship between *R_T_* and *l_G_* at room temperature and 550 °C, respectively. 2R_c_ is obtained by extrapolating the line fitted with concatenate fit method. The R^2^ value lies between 97–99%, thus confirms the fidelity of data. Ten data points are approximately used for each length of graphene. The linear fitting of data points for each graphene length successfully describes the implication of a method to measure the contact resistance R_c_. 

**Figure 9:**
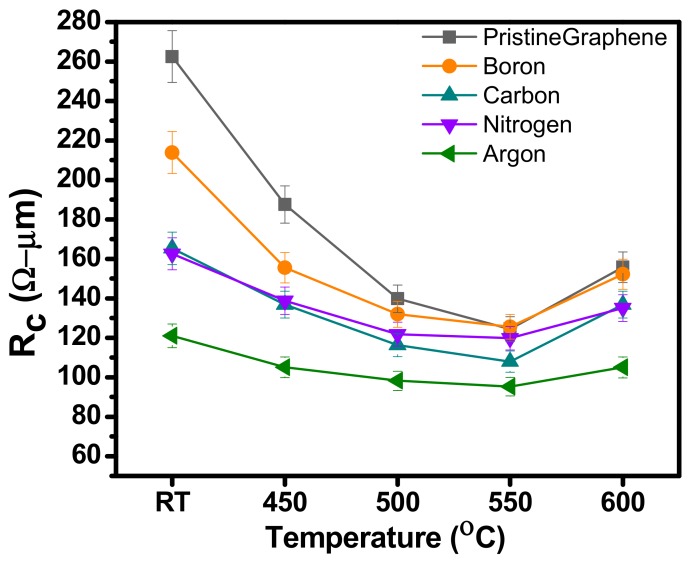
Specific contact resistance R_c_ of metal contact Pd/Au on pristine graphene and by irradiation of B, C, N_2_, and Ar ions for energy of 1.5 keV at the dose of 1 × 10^14^ ions/cm^2^ before and after RTA at 450 °C, 500 °C, 550 °C, and 600 °C.

Figure 9 shows the R_c_ values of Pd/Au contacts on B, C, N_2_, and Ar irradiated samples at an energy of 1.5 keV, and dose of 1 × 10^14^ ions/cm^2^. The minimum value obtained in the case of Ar ion irradiated sample i.e., 121.0 Ω-µm, while the maximum value achieved here is for B i.e., 213.8 Ω-µm before RTA. However, the N_2_ and C irradiated samples have R_c_ values of 162.5 Ω-µm and 165.2 Ω-µm, respectively. To gain more insight into the ion induced defect changes the R_c_ values of Pd on pristine graphene is also studied. The measured values are represented by grey line in the Figure 8 represented by legend pristine graphene. It is generally believed that Pd belongs to the chemisorbed family of metal contacts used, strong interactions are theoretically predicted [19]. Even though, it has been proved that carbon dissolution occurs without external influence during metal deposition on graphene grown by CVD, a metal graphene chemical reaction is expected to be more apparent at higher temperatures, thereby improving the contact of metal graphene [30]. The conductivity at the chemisorbed Pd-graphene interface is mainly controlled by the strong interaction between metal and graphene, where strong covalent bonds are formed, thus the conduction through graphene surface becomes insignificant. In order to find the best annealing conditions and to further improve the metal graphene contacts, the post-RTA was carried out at 450 °C to 600 °C with a step of 50 °C. Therefore, RTA is reported to be an improved method to reduce metal graphene contact resistance for different metal contacts. Figure 9 gives the detailed comparison of the R_c_ values brought about by the different RTA temperatures and shows the improved end contact formation through orbital hybridization at the intrinsic defected location of graphene.

Defect formation has been successfully established and also confirmed by the Raman spectroscopy results. This configuration provides shorter bonding sites with larger orbital overlap with respect to surface contacts. The first-principle quantum mechanical density functional and matrix Green’s function methods have shown that for edge-contacted M/G interfaces contact resistivity value decreases due to higher cohesive energy at the interface between the carbon atoms and the metal [29]. This also provides additional evidence to support the lower R_c_ value in the case of Ar.

Subsequently, defect yield by ion irradiation opens up the end contact regime for metal deposition. The defect concentration is large compared to other ions in the case of Ar irradiation. Therefore, the carbon dangling bond induced by defects hybridized with the chemically active metal d-orbitals. The density of states near Fermi energy E_f_ depends on the carbon p-orbital and metal d-orbital. However, for standard top contact, the carbon p_π_ orbitals contribute to the bonding of surface metals, and in the case of end contact, the P_σ_ orbitals also contribute to the bonding and transmission. In addition, the ion irradiation and ion species not only control the defects in this case, but also contribute to the realization of low contact resistance. This method provides a new way to induce defects for reducing contact resistance and improving M/G interface. Furthermore, there is a density of "gap states" in the hybridized region, which makes the graphene metallic rather than the “semi-metal”. The small equilibrium interface distance and a large number of interstitial gap states eliminate the tunnel wall barrier. Therefore, we conclude that M/G interface bonding is improved. The ion-induced defects inhibit carrier transport in metal-contacted graphene, and these defects do not form end-to-side contact without heat treatment. Therefore, with the ion irradiation the defects produced in the metal react with the dangling bonds and pave a way to minimize the current leakage from the edges.

After RTA treatment, the contact resistance of all samples decreased. The minimum value of R_c_ is 95.2 Ω-µm for Ar irradiated graphene with Pd/Au (40 nm/10 nm) contact. The RTA reported in this particular case is 550 °C. In Figure 8 we compared annealing effect on the R_c_ values of samples irradiated with B, C, N_2_, and Ar at energy of 1.5 keV and a dose of 1 × 10^14^ ions/cm^2^. The apparent decrease in resistance is attributed to the increased adhesion between metal and graphene. Furthermore, carbon dissolves into metals during RTA, which leads to so-called “end-to-end contact”, i.e., covalent bonds between metals and graphene, which reduces contact resistance. In case of annealing at 550 °C, we obtained a minimum R_c_ by irradiation of B, C, N_2_, and Ar ions for energy of 1.5 keV at the dose of 1 × 10^14^ ions/cm. This indicates that the higher annealing temperatures improves the end contact formation and enhance it to a certain limit. On the other hand, R_c_ value started to rise after RTA at 550 °C. The results showed in Figure 8 indicate that high-temperature RTA i.e., at 600 °C has adverse effect on contact resistance, possibly due to unintentional doping of graphene and strain effects induced due to annealing. In addition to this, it is expected that higher temperatures will be more effective in burning off organic residues left over during device fabrication. However, due to the increased coupling between the support substrate (i.e., SiO_2_) and graphene, it should be avoided that the temperature is higher than 600 °C. The explanation is consistent with the results provided in Figure 8. Obviously through such a behavior, the 600 °C is set to be the maximum annealing temperature in this experiment.

The lower value of R_c_ is obtained for Pd metal nearly equivalent to theoretical limits at room temperature and pressure conditions. The carrier injection efficiency between graphene and palladium is saturated because the graphene sample used is of high quality and has a long average carrier free path. The lower value of R_c_ achieved in this work is mostly attributed to the RTA and ion irradiation induced controlled defects. Furthermore, RTA helped the chemical bonding at the metal graphene interface. In this regard, the ion irradiation is successfully used to introduce defects in the CVD grown transferred graphene. Moreover, increased and uncontrolled defects in graphene may lead to carrier scattering, shorten the average free path of carriers, and increase contact resistance. The Ar ion energy 1.5 keV, dose 1 × 10^14^ ions/cm^2^ and 550 °C RTA temperature used in our work are favorable to produce a controlled amount of defects, which can be confirmed by the Raman spectroscopy. Therefore, the resulted R_c_ value achieved in this experiment is lower than previously reported values with Pd as metal contact. Moreover, from the above discussion, it is evident that RTA is more advantageous to improve the contact of metal graphene by enhancing the end contact formation of defects caused by ion irradiation. 

## 4. Conclusions

In this work we have demonstrated a novel method to improve the metal graphene interface and achieve a lower contact resistance with Pd metal contact. This is important for the realization of high-performance devices and integrated circuits. Specifically, we have introduced a new contact strategy to achieve low contact resistance using ion irradiated graphene defects. The mechanism of contact enhancement caused by annealing was studied systematically. We fabricated the residue free metal graphene contacts defined by the lithography on same transferred graphene flake. This enables us to fairly compare between the contact on pristine graphene and ion induced defected graphene. The comparable contact enhancement with the lowest value of R_c_ 95.2 Ω-µm achieved in the case of Ar irradiated graphene based fabricated devices at 550 °C RTA. The improved contact formation and lower R_c_ is carried out successfully by Ar ion irradiation. It is found that carbon dissolution in chemisorbed metal interface leads to many end contact formations between metal and carbon dangling bonds, which contributed in lower contact resistance. Furthermore, the results agree with the hypothesis that different ions could be used to significantly enhance the metal graphene interface by inducing defects. It has been estimated by Raman spectroscopy that ion defects produced by Ar and N_2_ ions are larger in size due to their mass. In addition, extensive experimental insight of contact properties of Pd to graphene modified by ion induced defect is provided. The ion induced defects effect on contact properties were established before and after RTA. In short, the results showed that ion-induced defects before metallization and RTA treatment provide a convenient method for reducing contact resistance.
